# Demographic, social, and clinical aspects associated with access to COVID-19 health care in Pará province, Brazilian Amazon

**DOI:** 10.1038/s41598-024-59461-1

**Published:** 2024-04-16

**Authors:** Amanda Loyse da Costa Miranda, Ana Rosa Tavares da Paixão, Andrey Oeiras Pedroso, Laís do Espírito Santo Lima, Andressa Tavares Parente, Eliã Pinheiro Botelho, Sandra Helena Isse Polaro, Ana Cristina de Oliveira e Silva, Renata Karina Reis, Glenda Roberta Oliveira Naiff Ferreira

**Affiliations:** 1 Programa de Pós-Graduação Em Enfermagem, Universidade Federal Do Pará, Belém, 66075-110 Brasil; 2 Escola de Enfermagem de Ribeirão Preto, Universidade de São Paulo, Ribeirão Preto, 14040-092 Brasil; 3 Programa de Pós-Graduação Em Enfermagem, Universidade Federal da Paraíba, João Pessoa, 58051-900 Brasil

**Keywords:** Health services, Infectious diseases, Viral infection

## Abstract

Internal social disparities in the Brazilian Amazon became more evident during the COVID-19 pandemic. The aim of this work was to examine the demographic, social and clinical factors associated with access to COVID-19 health care in Pará Province in the Brazilian Amazon. This was an observational, cross-sectional, analytical study using a quantitative method through an online survey conducted from May to August 2023. People were eligible to participate if they were current residents of Pará, 18-years-old or older, with self-reported diagnoses of COVID-19 through rapid or laboratory tests. Participants completed an electronic survey was developed using Research Electronic Data Capture (REDCap) software—The adapted questionnaire “COVID-19 Global Clinical Platform: Case Report Form for Post-COVID Condition”. Questions focused on access to COVID-19 treatment, demographic characteristics, COVID-19 vaccine and clinical characteristics. Respondent-driven sampling was applied to recruit participants. Multiple logistic regression was utilized to identify the associated factors. Overall, a total of 638 participants were included. The average age was 31.1 years. Access to COVID-19 health care was 68.65% (438/638). The participants most likely to access health care were those with moderate or severe COVID-19 (*p* = 0.000; OR: 19.8) and females (*p* = 0.001; OR: 1.99). Moreover, participants who used homemade tea or herbal medicines were less likely to receive health care for COVID-19 in health services (*p* = 0.002; OR: 0.54). Ensuring access to healthcare is important in a pandemic scenario.

## Introduction

COVID-19 was declared a pandemic in March 2020 and has become one of the greatest global health crises of the last hundred years^1–2^. In Brazil, the infection and disease affected thousands of people, with deaths recorded in all states of the federation, including the northern region located in the Brazilian Amazon^3^.

During the COVID-19 outbreak, despite accounting for only 7.73% of the confirmed cases in Brazil in the Brazilian Amazon region, the population was disproportionately impacted by COVID-19; this was primarily attributed to the area's sociodemographic composition, inadequate health care services, barriers to geographic mobility, deficient transport infrastructure, and lack of preparedness in coordinating an effective response to the COVID-19 pandemic^3–8^. Throughout this time period, internal social disparities in the region became more evident. Studies have indicated that the most affected groups included marginalized individuals with low income and education who lacked adherence to mask usage and social distancing. Moreover, indigenous refugees, those with limited health care accessibility, and individuals resorting to self-medication for prevention were notably affected^9–11^; this indicates that the majority of the population is dependent on the Unified Health System (Sistema Único de Saúde, acronym SUS, in Portuguese), with universal access to health care services and cost-free access across all levels, thus emphasizing the significance of primary health care (PHC) as the primary entry point for this system^12^.

The population was affected by the pandemic and problems in the national management of the public health system during this crisis, which affected the diagnosis and treatment of COVID-19 patients. Management failures included the indication of ineffective medications for the treatment of COVID-19, such as chloroquine and ivermectin, and interference with the national vaccination plan against the virus^13^. In this context, local governments coordinated local initiatives to organize the health system. Regarding system management, collaboration with universities was fundamental, thus enhancing the use of epidemiology as a management tool and facilitating access to laboratory services and hospital care^5^.

Notably, as a consequence of the previously described scenario, the Amazon region experienced a serious collapse in its local health care system, which was especially evident during the second wave of COVID-19 in January 2021 in Manaus, Amazonas; this was represented by hospitals lacking an oxygen supply^16–18^. This catastrophic situation likely led to underreporting of COVID-19 infections, as many individuals (particularly those who did not access health care) remained untested^18^. Extensive studies have investigated various aspects related to COVID-19 in the Amazon context. However, there remains a gap in the understanding of the factors associated with access to health care, specifically for COVID-19-related health care^9–11^.

In the scenario in which demographic and clinical factors interact with other factors and influence access to health care, a framework for the study of access to health care was adopted^19^. Scholars have investigated access to health care for other illnesses not related to COVID-19^20–22^. Only one study has been conducted to examine geographic access to COVID-19 health care in Brazil and revealed that the amount of equipment available varies between municipalities and is lower among black and poor communities^22^.

This study is especially crucial in scenarios characterized by limited availability of health care^8,23–24^, geographic challenges^6^ and inadequate management of the pandemic^13,15,18^. Therefore, the aim of this work was to examine the demographic, social and clinical factors associated with access to COVID-19 health care in Pará Province in the Brazilian Amazon; this particular state stands out as having the highest concentration of COVID-19 cases among North Region states (accounting for 27.81% of cases), with its capital (Belém) serving as the epicenter of the pandemic within this state^3^.

## Methods

### Study design and setting

This was an observational, cross-sectional, analytical study using a quantitative method through an online survey conducted from May to August 2023.

**Figure 1:**
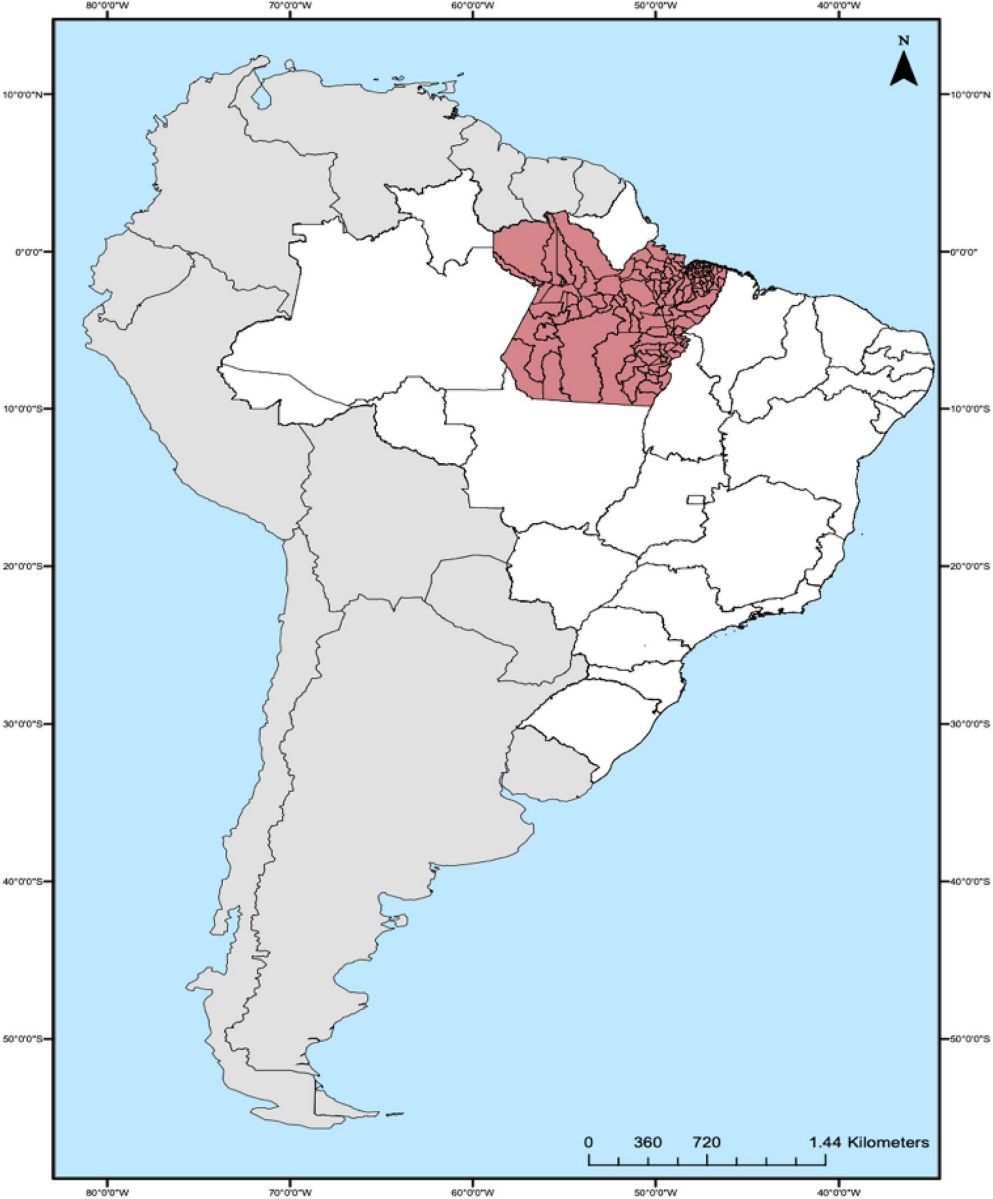
The location of the state of Pará © 2023 by Amanda Miranda (software ArcGIS 10.8, https://www.arcgis.com/index.html) is licensed under CC BY-ND 4.0. To view a copy of this license, visit.

The Pará Province includes 144 municipalities, with 1,245,870.704 square kilometers, located in the Brazilian Amazon in the northern region of Brazil (Fig. 1); additionally, it has an estimated population of 8,777,124 inhabitants. This population exhibits precarious social and health indicators. In 2022, 1,808 PHC teams were registered to serve 5,391,499 inhabitants. This low coverage of PHC reduces access to other levels of the health system, when considering that in Brazil, the PHC coordinates care^25–26^.

### Participants and selection criteria

People were eligible to participate if they were current residents of Pará, 18-years-old or older, with self-reported diagnoses of COVID-19 through rapid or laboratory tests. The exclusion criterion included people who did not have access to the internet or electronic devices to answer the questionnaire.

### Study size

The calculations were performed in the population survey in the EpiInfo version 7.2.5.0 in the StatCalc module. For the sample calculation of the quantitative study, the following parameters were adopted: 777,901 people with COVID-19 infection in Pará; margin of error of 5%; 95% confidence interval, 38.6% frequency of people with reactive IgG who used preventative medication for COVID-19^9^ (people who received preventive medication for COVID-19, including medicines that require a doctor's prescription – people who sought health care to treat COVID-19). The use of health care was not limited to people with moderate or severe symptoms, thus resulting in a minimum sample of 364 participants. This number quickly exceeded that of the online survey; therefore, it was decided to use the total number of people who responded to the questionnaire during the data collection period.

### Study instrument (questionnaire)

To collect data, an adapted version of the “Global COVID-19 Clinical Platform: Case Report Form for Post-COVID-19 conditions” questionnaire from the World Health Organization was used^27^. The questionnaire (structured) was adapted and translated by researchers from three federal universities in different regions of Brazil to make the language clear and accessible to the general population. To make the questionnaire easy to complete, unnecessary reading effort was reduced, and a cohesive and coherent order was established between the questions. Participants took an average of 15 min to complete the questionnaire. The questionnaire was organized into six domains. For this study, only two domains were used: sociodemographic and clinical characteristics related to SARS-CoV-2 infection.

In the sociodemographic domain, the following variables were used: sex (female/male); traditional population (yes/no); age (years); skin color (black/brown/indigenous/white/yellow); education level (did not study/elementary/high school/university); living in the state capital (yes/no); current job (unemployed/student/retired/employed/self-employed); beneficiary of social programs (yes/no); tobacco use during the pandemic (yes/no); use of alcoholic beverages during the pandemic (yes/no); personal monthly income minimum wage (in reais) (equal or less than 1 salary/above 1 salary); marital status (married/common-law/divorced/separated/widowed/single).

In the clinical characteristics domain related to SARS-CoV-2 infection, the following variables were considered: number of COVID-19 reinfections that were laboratory confirmed (mean); year of first COVID-19 infection (2020–2021/2022–2023); chronic illness before COVID-19 infection (yes/no); and classification of the most serious COVID-19 infection (severe: was admitted to the intensive care unit or needed to be intubated/moderate: had proven pneumonia or was hospitalized or needed oxygen support/mild: respiratory symptoms, but no pneumonia or shortness of breath); herbal medicines and homemade tea (yes/no); COVID-19 vaccine (yes/no); number of doses of COVID-19 vaccine (mean); and COVID-19 infection before being vaccinated (yes/no).

In this study, we considered traditional populations of the Amazon as those inhabiting the waters, fields, and forest: quilombola remnants (Afro-descendants who live in specific territories), indigenous peoples and riverside dwellers, following the concept established by the National Policy for Comprehensive Health of Rural and Forest Populations^28^.

### Data collection

Participants completed an electronic survey using Research Electronic Data Capture (REDCap)® –software, including the adapted questionnaire “COVID-19 Global Clinical Platform: Case Report Form for Post-COVID-19 Condition”. Respondent-driven sampling (RDS), which is a variant of the "snowball sampling" methodology that is widely used in online surveys, was applied to recruit participants^29–30^. Through this method, participants are encouraged to share the questionnaire link with other individuals who meet the study's inclusion criteria to respond to the survey via social networks, with WhatsApp® being the social network that was used in this study.

To reach the minimum sample size, students from health-related undergraduate programs in all of the municipalities of the state were recruited. They underwent a four-hour training session on conducting the online survey by using the RDS methodology and the clarification of questionnaire-related queries. Each student invited 10 individuals from their circle who reported of COVID-19 infection to participate in the research. For each respondent who completed the survey, they correspondingly referred another 10 individuals, thus creating reference chains that increased the recruitment cycles/waves and expanding the number of participants^29–30^. In this study, 201 students were recruited and divided among 31 training sessions. Each student recorded data in an Excel spreadsheet, which included the number of participants who they invited, how many people were referred by each invitee, and other factors. This process was repeated until researchers achieved the minimum sample for the study.

The completed questionnaires were hosted on the REDCap® platform, which is designed for online data management and features settings and tools that enhance organizational capacity and security for storing information. It is important to highlight that, given the nature of the online survey, all of the collected information was self-reported.

### Variables

The response variable of the study was the question “How was your treatment/health care?”. The answers to this question were “I was treated alone, without evaluation by a health care professional”, “I was admitted to a ward”, “I was admitted to an intensive care unit”, “I was treated at home, with support from health care professionals via telephone or internet”, “I received care in an outpatient clinic (the clinics provide specialized outpatient secondary care)”, “I received care in a private hospital (agreement/health insurance)”, “I received care in an emergency room or an emergency care unit”, and “I received care in a health center (PHC)”. The health center or basic health unit involves primary health care services provided by generalist health professionals, which can be offered by the government (free) or in private services. In Brazil, there are a large number of services offered by the government.

All of the responses in which participants stated that they received health care were grouped into a single response: “I received health care”. The outcome is a dichotomous qualitative variable, “Received” or “Not Received (self-care)”. The response event was "received". The independent variables included the variables that were included in the questionnaire, which are divided into the following domains: demographic, social characterization, and clinical characteristics.

### Quantitative variables

The variables age, number of COVID-19 reinfections, and number of doses of the COVID-19 vaccine were analyzed as quantitative (continuous variables) independent variables.

### Statistical methods

The data were extracted from REDCAP® and exported to Microsoft Excel®. Descriptive statistics, means, standard deviations, confidence intervals, absolute frequencies, and percentages were calculated. The result was presented by using texts, graphics, and tables. The 'nonresponse” variables were excluded from the statistical analysis, and no percentage was calculated.

The main hypothesis of the study, “factors associated with receiving treatment/health care (access to health care)”, was tested by using multiple logistic regression. When considering the dichotomous nature of the response variable, binomial regression was initially used to assess the association between each independent variable and the dependent variable. All of the variables were tested in this model, and variables with a p value < 0.020 were selected for inclusion in the multiple logistic regression model (sex, traditional population, age, education level, living in the state capital, individual income, marital status, number of COVID-19 reinfections, chronic illness before COVID-19 infection, classification of the most serious COVID-19 infection, use of herbal medicines and homemade tea, and number of doses of COVID-19 vaccine).

In the final multiple logistic regression model, the variables with p values < 0.20 were analyzed together by using the backward elimination model. This involves the introduction of all factors that were selected in the first stage and at each stage of the regression and is automatically removed by the program until only factors with a p value < 0.10 remain (number of COVID-19 reinfections, sex, chronic illness before COVID-19 infection, classification of the most serious COVID-19 infection, and use of herbal medicines and homemade tea).

All p values < 0.05 were considered to indicate statistical significance. To interpret the results, quality tests, coefficient values of the independent variables, Z values, 95% confidence intervals, and odds ratios were considered in the regression. The analyses were performed by using the Bioestat 5.3®, Microsoft Excel®, and Minitab 20® programs.

### Ethics approval and consent to participate

All of the requirements proposed by Resolution 466 of 2012 of the National Health Council of Brazil were followed, as well as all of the principles established in General Personal Data Protection Law No. 13,709 of 2018 concerning personal data processing activities, as established in articles 6 and 7. The Declaration of Helsinki was followed. The study was approved by the Research Ethics Committee of the Federal University of Paraiba Lauro Wanderley University Hospital under protocol number 5.826.893 and CAAE: 65929522.1.0000.5183. All of the participants signed the Free and Informed Consent Form. The authors did not perform experiments on humans and/or use human tissue samples/human data. The authors did not have direct contact with the study participants because the study questionnaire was administered via the internet, and the data were stored by REDCAP®, which guarantees security in terms of privacy and data storage.

## Results

### Sociodemographic characteristics

In this study, 438 (68.65%) out of 638 participants received health care for COVID-19, whereas the remaining 200 (31.35%) participants chose self-care (not received). The average age was 31.1 years (standard deviation [SD] = 11.9; 95% CI: 30.2, 32.0), and the 18- to 32-year-old age group had the highest percentage at 66.3% (421). Among the participants, 68.3% (436) were female; 40.8% (258) were employed or retired; 35.9% (229) were self-declared students; 57.9% (357) had incomes less than or equal to the minimum wage; 74% (472) were unrelated (divorced, separated, widowed or single); 11.7% (74) were beneficiaries of social programs; and 3.5% (22) were people belonging to traditional populations (Table 1).

**Table 1:** Associations between sociodemographic characteristics and access status to COVID-19 health care Pará. Brazil. 2023.

Social and demographic characteristics	Access to COVID-19 health care (effective utilization of the services)			Regression				
	Not received (self-care) (200) n (%)	Received (438) n (%)	Total (638) n (%)	COR		(95% CI)	*p* value	
Sex								
Female	122 (28)	314 (72)	436 (68.3)	1.62		(1.38; 2.30)	0.007	
Male	78 (38.6)	124 (61.4)	202 (31.7)			Ref		
Traditional populations								
Yes	3 (13.6)	19 (86.4)	22 (3.5)	3.03		(0.88; 10.3)	0.07	
No	196 (32.4)	409 (67.6)	605 (96.5)			Ref.		
NI	1	10	11					
Age range								
Average (SD)	29.1 (10.7)	31.7 (12.3)	31.1 (11.9)	1.02		(1.00; 1.03)	0.01	
18–32	147 (34.9)	274 (65.1)	421 (66.3)					
33–47	38 (26.6)	105 (73.4)	143 (22.5)					
> 47	15 (21.1)	56 (78.9)	71 (11.2)					
NI		3	3					
Skin color								
Black/Brown/Indigenous	60 (29.6)	143 (70.4)	203 (31.8)	0.88		(0.61; 1.27)	0.50	
White/yellow	140 (32.2)	295 (67.8)	435 (68.2)			Ref.		
Education Level								
No education/elementary school/middle school	70 (36.8)	120 (63.2)	190 (29.8)			Ref.		
University	130 (29.0)	318 (71.0)	448 (70.2)	1.42		(0.99; 2.04)	0.05	
Residence in the capital city								
Yes	75 (28.0)	193 (72.0)	268 (43.0)		Ref.			
No	123 (34.6)	232 (65.4)	355 (57.0)	1.36	(0.96; 1.92)			0.07
NI	2	13	15					
Current job								
Unemployed/Student/Retired/	107 (33.9)	209 (66.1)	316 (49.9)		Ref.			
Employed/Self-employed	93 (29.3)	224 (70.7)	317 (50.1)	1.23	(0.88; 1.72)			0.22
NI	0	5	5					
Beneficiary of social programs								
No	177 (31.8)	379 (68.2)	556 (88.3)		Ref			
Yes	22 (29.7)	52 (70.3)	74 (11.7)	1.103	(0.65; 1.87)			0.71
NI	1	7	8					
Tobacco use during the pandemic								
Yes	36 (36.7)	62 (63.3)	98 (16.0)	0.75	(0.47; 1.18)			0.21
No	157 (30.4)	359 (69.6)	516 (84.0)		Ref.			
NI	7	17	24					
Use of alcoholic beverages during the pandemic								
Yes	119 (30.8)	267 (69.2)	386 (62.6)	1.05	(0.74; 1.50)			0.75
No	74 (32.0)	157 (68.0)	231 (37.4)		Ref			
NI	7	14	21					
Personal monthly income *minimum wage (in reais)								
Equal or less than 1 salary	122 (34.2)	235 (65.8)	357 (57.9)	0.76	(0.54; 1.08)			0.13
	74 (28.5)	186 (71.5)	260 (42.1)		Ref.			
NI	4	17	21					
Marital status								
Married/Common-law marriage	44 (26.5)	122 (73.5)	166 (26.0)		Ref.			
Divorced/separated/widowed/single	156 (33.1)	316 (66.9)	472 (74.0)	0.73	(0.49; 1.08)			0.11

*COD* crude odds ratio, *CI* confidence intervals, *No* Information, *Ref*. reference, *SD* standard deviation.

*Minimum wage in Brazil in May 2023—R$ 1.320.00 (US$ 260,15—quotation for the same period).

Table 1 presents the results of the binary regression of the associations between sociodemographic characteristics and access status to COVID-19 health care in Pará Province in the Brazilian Amazon region. The results demonstrated that female participants were 1.62 times more likely to access/receive COVID-19 health care than male participants were (p = 0.007). As age increased, the chances of accessing/receiving COVID-19 health care also increased (p = 0.01; OR: 1.02). Variables with p values < 0.20 were selected for multiple regression, including traditional population, educational level, lives in the state capital, individual income, and marital status.

### Clinical characteristics

**Figure 2:**
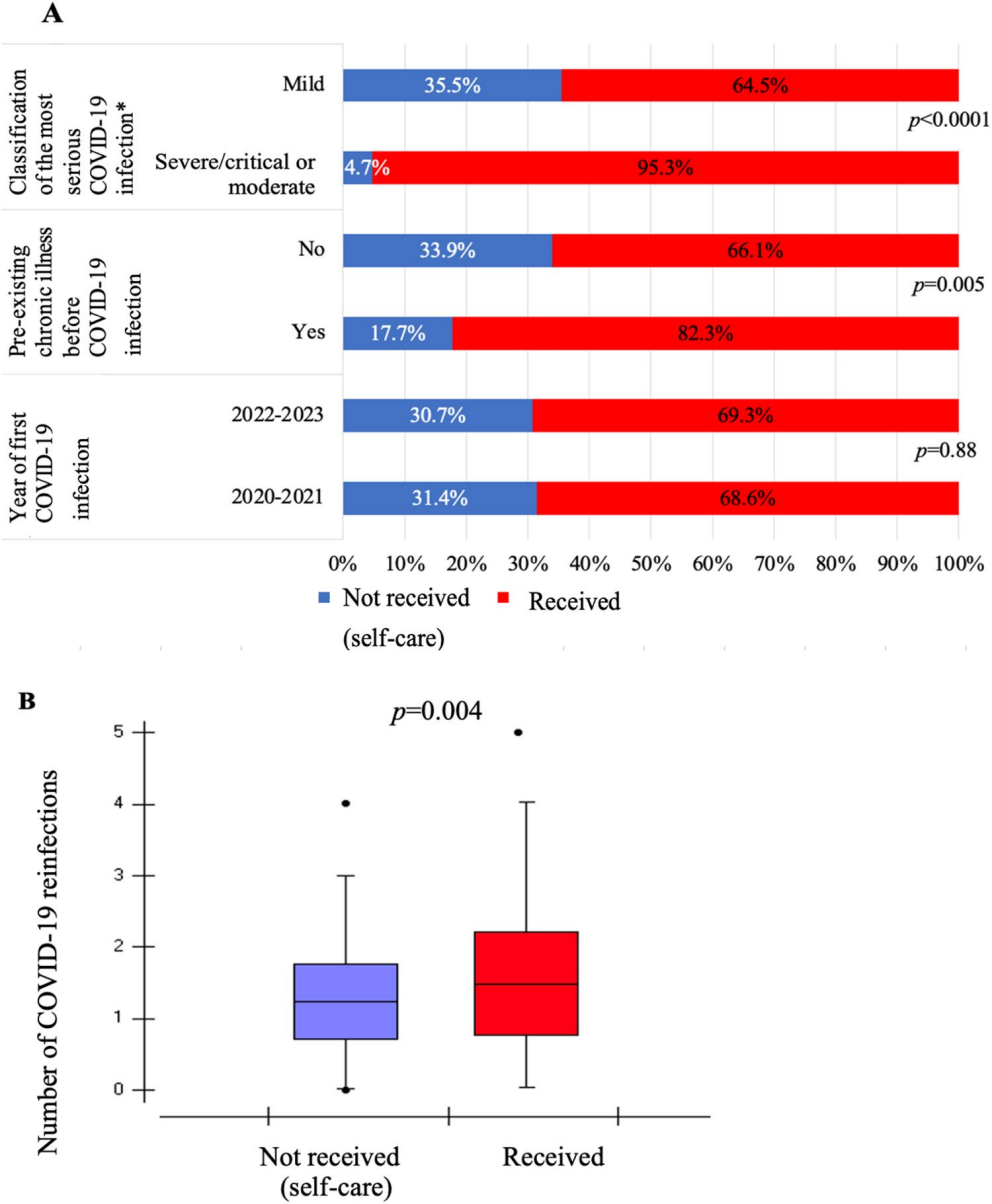
Associations between clinical characteristics, COVID-19 reinfections and access status to COVID-19 health care. Figure 2A—Year of diagnosis, diagnosis of chronic disease and classification of infection*. 2B—Number of COVID-19 reinfections (average). Pará. Brazil. 2023. *Classification of the most severe COVID-19 infection (Severe: was admitted to the intensive care unit or needed to be intubated/Moderate: had proven pneumonia or was hospitalized or needed oxygen support/Mild: respiratory symptoms, but no pneumonia or shortness of breath).

Figure 2 shows the clinical characteristics and COVID-19 infection status of the patients. Regarding the year of first COVID-19 infection, 82% (519) of participants were diagnosed between 2020 and 2021, and 18% (114) of participants were diagnosed between 2022 and 2023. Only five (5) participants did not respond. The first year of COVID-19 infection was not associated with access to COVID-19 health care (p = 0.88; OR: 0.96; 95% CI: 1.13, 1.95). Regarding the presence of any preexisting chronic conditions before COVID-19 infection, among the 598 participants who responded to this inquiry, 86.8% (519) reported of no history of chronic ailments, whereas 13.2% (79) acknowledged of having some form of preexisting disease. Participants with a chronic disease were 2.38 times more likely to access health care than were those without any disease (p = 0.005; 95% CI: 1.30, 4.36). The majority of participants at 86.5% (552) were classified as having mild COVID-19 infection, whereas only 13.5% (86) had severe or moderate infections. Individuals displaying moderate or severe COVID-19 infection had an 11.2-fold greater likelihood of receiving health care than those exhibiting mild symptoms of the disease (p < 0.0001; 95% CI: 4.07, 31.2) (Fig. 2A).

Figure 2B illustrates the number of COVID-19 reinfections based on the utilization of health care. Among individuals who did not access health care (self-care), the mean number of reinfections was 1.29 (SD: 0.66), whereas among participants who received health care, the average was 1.47 (SD: 0.71) reinfections. The regression analysis demonstrated that the likelihood of receiving health care increased according to the number of COVID-19 reinfections (p = 0.004; OR: 1.48; 95% CI: 1.13, 1.95).

**Figure 3:**
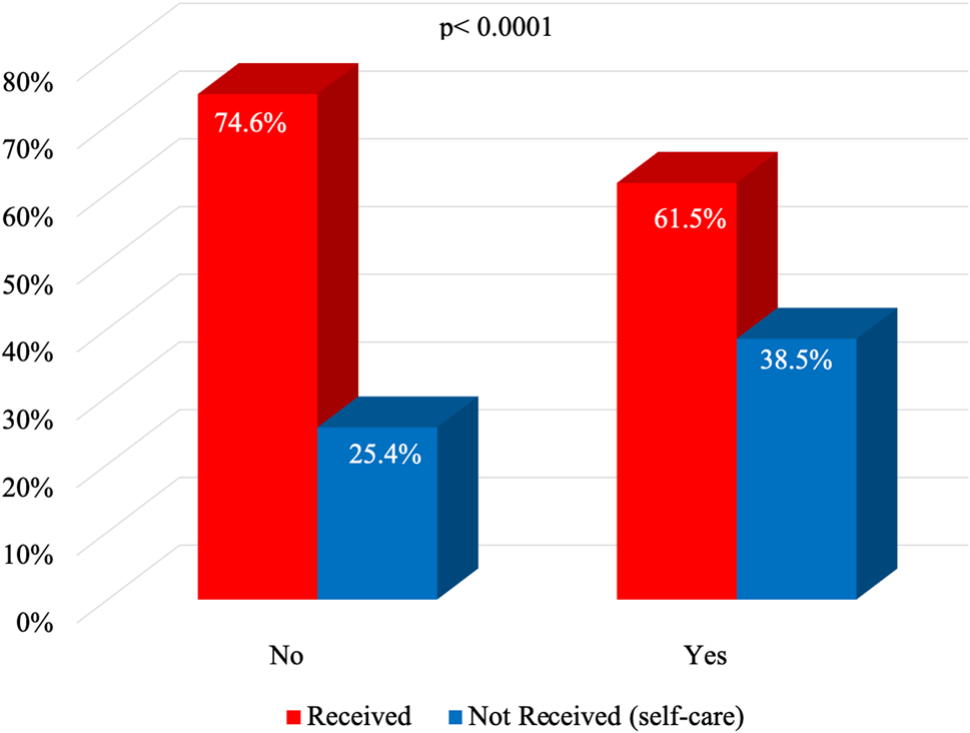
Association between herbal medicines/homemade tea and access to COVID-19 health care. Pará. Brazil. 2023.

Figure 3 shows the association between the use of natural/alternative medications to treat COVID-19 and access to health care. Among these individuals, 631 participants responded to the inquiry regarding the utilization of herbal medicines and homemade tea, and 54.8% (347) chose not to use them, whereas 45.2% (286) reported of using these remedies. Individuals who used homemade tea or herbal remedies (p < 0.0001; OR: 0.54; 95% CI: 0.38, 0.76) were less likely to receive health care.

**Figure 4:**
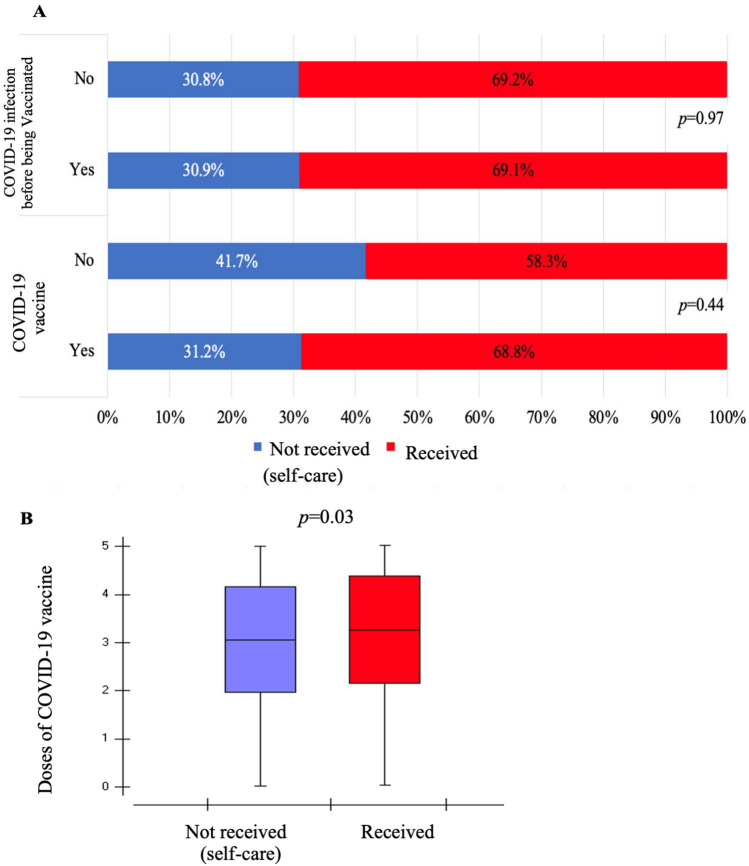
Association between COVID-19 vaccines and access to COVID-19 health care 4A—COVID-19 infection before being vaccinated and after receiving the COVID-19 vaccine. 4B—Number of doses of the COVID-19 vaccine. Pará. Brazil. 2023.

Figure 4 presents the association between the COVID-19 vaccine and access to health care. Regarding the question about having received the COVID-19 vaccine, 1.9% (12/629) did not receive it, of whom 58.3% (7) received COVID-19 health care and 41.7% (5) did not have access to health care. Among the 98.1% (619/629) who received the vaccine, 68.8% (426) received health care, and 31.2% (193) received self-care (4A). Regarding having a COVID-19 infection before being vaccinated, 25.5% (156/610) received the vaccine before COVID-19 infection, of whom 69.2% (108) received health care. Moreover, among the 74.5% (456/610) who had COVID-19 infection before receiving the vaccine, 69.1% (315) received health care. There was no association between these variables and access to health care (Fig. 4A).

Figure 4B displays the number of vaccine doses according to access to health care. A total of 615 participants answered the survey, with an average of 3.3 vaccine doses (SD: 0.94). Among those who did not access health care (self-care), the average was 3.18 vaccine doses (SD: 0.92), whereas the average was 3.35 doses (SD: 0.94) among participants who received health care. The regression analysis demonstrated that the likelihood of receiving COVID-19 health care increased with the number of COVID-19 vaccine doses (p = 0.03; OR: 1.22; 95% CI: 1.01, 1.46).

### Sociodemographic and clinical characteristics: multiple logistic regression model

In the final model (Table 2), all of the variables that were selected in the previous step were analyzed together. The participants most likely to access health care were those with moderate or severe COVID-19 (*p* = 0.000; OR: 19.8) and females (*p* = 0.001; OR: 1.99). Moreover, participants who used homemade tea or herbal medicines were less likely to receive health care for COVID-19 in health services (*p* = 0.002; OR: 0.54).

**Table 2:** Multiple logistic regression analysis of the associations between social, demographic, and clinical characteristics and access status to COVID-19 health care. Pará. Brazil. 2023.

Social, demographic, and clinical	*P*	AOR*	(95% CI**)
Number of COVID-19 reinfections	0.053	1.35	(0.99; 1.83)
Sex (ref.: male)			
Woman	0.001	1.99	(1.33; 3.00)
Chronic illness before COVID-19 infection (ref.: no)			
Yes	0.064	1.83	(0.96; 3.48)
Classification of the most serious COVID-19 infection (ref.: mild)*			
Severe/critical or moderate	0.000	19.8	(4.75; 83.0)
Use of herbal medicines and homemade tea (ref.: no)			
Yes	0.002	0.54	(0.36; 080)

*AOR* adjusted odds ratio, *CI* confidence intervals, *Ref.* reference.

*Severe (was admitted to the intensive care unit or needed to be intubated), Moderate (had proven pneumonia or was hospitalized or needed oxygen support) and Mild (had respiratory symptoms, but no pneumonia or shortness of breath).

## Discussion

In this study, the concept of access to health services was used to examine the realized access (effective use of services)^19^ and associated factors^19,21–22^. The results indicated that the individuals most likely to access COVID-19 health care were females and those who had moderate or severe COVID-19 infections. However, participants who used homemade tea or herbal medicines were less likely to access COVID-19 health care.

A high proportion of participants had access to COVID-19 health care. This result may be related to the characteristics of the sample but also to the universal health system in Brazil, which has a large capillarity through the PHC^12^.

This study considered the type of physical access in establishments of public and private health networks at all levels of health care and virtual access. The characteristics of the population are important aspects in studies on access, and such aspects can facilitate or be barriers to access^19^. In this study, the predisposing factors included education, age, ethnicity (traditional population), current job, marital status, and sex. The enabling factors included income, participation in social programs, residence in the capital city, and availability of health services such as vaccines, including doses and COVID-19 diagnosis. Need factors^19^ included chronic conditions, the use of alcohol and tobacco, and mild to severe COVID-19.

### Sociodemographic characteristics

The state of Pará encompasses several municipalities characterized by the poorest income, employment, and social indicators in Brazil^25–26^, thus leading to a disproportionate impact of COVID-19 on its population, particularly affecting vulnerable people^10–11^. The province of Pará has several cities characterized by the lowest income, employment and social indicators in Brazil^25–26^. These characteristics of the social and health structure are aspects that increase the population's risk for COVID-19 infection and its outcomes, particularly affecting vulnerable individuals^10–11^.

The specific characteristics of these municipalities and their populations played a significant role in both the spread of cases and the accessibility of health services during the pandemic. In these poor areas, residents experienced higher mortality rates from COVID-19. The failure to collect diagnostic samples was 3.07 times more frequent in these locations, and there was a 140% shortage in the availability of computed tomography scans^31^. A study conducted in Brazil highlighted an increased death rate among younger age groups in the most deprived municipalities, especially among individuals under 40-years-old who belonged to indigenous backgrounds. This observation led to the conclusion that levels of deprivation significantly hindered the prompt referral of patients to appropriate care^32^.

In the present study, multiple logistic regression analysis demonstrated that among the sociodemographic variables that were examined, only sex exhibited statistical significance within the final regression model. Notably, despite including other variables such as age, traditional population, educational level, residence in the capital, individual income, and marital status in this analysis, only sex was statistically significant.

Contrary findings have been observed in studies conducted across the Amazon region^9–11^. These studies, which were conducted during the initial and subsequent waves in Belém^10^, among the Waraó indigenous community^11^ and in the population of Manaus^9^, did not establish a clear association between sex and the incidence of COVID-19. However, in Germany, there was an identified correlation between COVID-19 and male sex, particularly among symptomatic individuals who accessed care at health care facilities^33^. Similarly, in Australia, men display a greater risk of hospitalization than women^34^. In contrast, a study in Jordan reported that the majority of hospitalizations were among women^35^.

In Brazil, a study examining the risk factors associated with hospitalization and mortality due to demographic, clinical, and socioeconomic variables demonstrated that, in general, males exhibited greater odds of death than females did. However, postpartum patients who were admitted to hospital wards were found to have increased odds of mortality^32^.

Access to health care services by women is a facet of health system performance that goes beyond mere availability; it is contingent upon the effective utilization of these services. This access is influenced by various factors, including the societal roles and rights attributed to women, cultural and religious norms, and the structure of health care financing models^36–37^. A specific study identified several contributing factors to the lower access of women to health care services, COVID-19 levels of women's empowerment, financial access, and educational opportunities. This disparity has led to the underreporting of COVID-19 cases and fatalities among women^37^.

In Brazil, the universal model of the health care system, which offers free access to the entire population, serves as a mechanism to alleviate the barriers imposed by the necessity of payment for health care services^12^. Notably, women in this country tend to visit health services more frequently than men, whether for preventative treatments or nonchildbirth-related hospitalizations. Urban areas demonstrate greater coverage by health plans for women, whereas in rural regions, women tend to rely more on the public health system, and men tend to allocate more of their resources to healthcare^38–39^. An understanding of the exacerbation of sex inequalities during pandemics is crucial. Within a pandemic context, among various factors, the inaccessibility of health services can significantly heighten women's vulnerabilities during a crisis^40–41^. Multiple analyses of children with COVID-19 infection demonstrated that being under 10 years of age, being a black or mixed race/ethnicity, and having a health condition prior to COVID-19 were associated with a greater likelihood of hospitalization^42^.

The sample had a greater number of participants with a high level of education; specifically, most participants had a lower income, and 35.9% were currently studying. A high rate of access to health services was also found in a study with a sample of participants with high education and income. However, it was observed that a preexisting relationship with a primary care provider was critical to receiving access to a health care provider and medication during the COVID-19 pandemic^21^.

However, vaccination against COVID-19 and the number of doses were not significant in the final regression model. There is a high percentage of vaccinated people. This result may be related to the vaccination strategy that was adopted in the country, which began with priority groups and later expanded to the entire population. In this country, this specific protection measure has universal and free access, which is financed by the government. The history of vaccine acceptance in Brazil and mandatory vaccination for COVID-19 led to 70% coverage in 2021 and approximately 60% coverage in the Northernregion^30^.

### Clinical characteristics

In the current study, individuals demonstrating moderate or severe clinical manifestations of COVID-19 were more inclined to access health care. Previous research has indicated that systemic and pulmonary clinical manifestations are linked to hospitalizations^35,43^.

The clinical manifestations of COVID-19 infection are needed. The availability of resources, which represents an enabling factor, is closely related to the need factor^19^. This effect was also observed in a study performed in the United States of America, which demonstrated that demographic factors, such as ethnicity and language, were associated with reduced access to health care, more severe COVID-19 infection on admission and mortality. A high proportion of Hispanic participants who did not have a health record prior to hospitalization were observed compared to non-Hispanics^44^. A previous study demonstrated that among those hospitalized for COVID-19, a lack of health insurance also impacted the use of resources, such as oxygen and physiotherapy, for treatment and care after hospital discharge^45^.

Geographic accessibility also contributes to the utilization of health services. In severe COVID-19 infections, the type of health service and the availability of physical resources for treatment have a direct impact on access^22^. Addressing individuals with severe and moderate clinical manifestations of COVID-19 necessitates adequate infrastructure, essential equipment, supplies, and a competent cadre of health care professionals^35,43–45^. However, within the context of the present study, the northern region had the lowest number of beds per thousand inhabitants in Brazil during the COVID-19 pandemic. Despite this, it experienced the most significant average increase in intensive care unit beds across the country. Among the 27 Brazilian states, Pará ranked sixth for the state with the lowest number of intensive care unit beds and third for other beds, with 0.15 and 1.87 beds per 1,000 inhabitants, respectively. Additionally, it registered the lowest number of nurses per thousand inhabitants (1.03) in Brazil, and the Amazon region experienced the lowest number of medical doctors (2.02 per 1,000 inhabitants)^31^. The distribution of increased resources, such as beds and mechanical ventilators, was unequal in the Brazilian Amazon region and within states, with urban areas and capitals having more abundant access to these resources^5,43,46^. These disparities in health care provision significantly impact access to health care services^36^.

Acute COVID-19 infection, similar to chronic disease, does not depend on continued care over time. It is an acute, episodic infection for which resources are available for immediate treatment^42–45^. A previous study estimated that 41% of the vulnerable population in 20 cities of Brazil, including Belém, lives farther than 5 km from a health service to admit patients in severe condition due to COVID-19 infection. Health services with intensive care unit beds and ventilators, which are essential for treating severe COVID-19 infections, are more precarious in the peripheral areas of cities^22^.

This study demonstrated that the utilization of teas and herbal medicines was linked to a reduced likelihood of accessing health care services for the treatment of COVID-19. Inhabitants of the Amazon region regularly incorporate homemade teas and herbal remedies derived from local plants into their daily lives. These plants are utilized for various therapeutic purposes, thereby addressing conditions such as microbial infections, gastrointestinal disorders, and inflammation^47^. Similar practices are occurring globally, with populations elsewhere using medicinal plants for disease prevention and increasing consumption postdiagnosis in an effort to alleviate symptoms^48^. An understanding of the active ingredients present in plants that are used for therapeutic purposes is pivotal^49^. In this study, individuals exhibiting severe or moderate clinical manifestations also required and accessed health care within a health service. Therefore, identifying individuals or populations who are most in need of health care services is crucial for assisting in the preparation of health care network management and care protocols. Studies on access to health care have described the care provided by specialist physicians, nurses, general physicians, and health workers. The care provided by traditional healers was not described in these previous articles^20^.

### Strengths and limitations

The utilization of the electronic questionnaire may have restricted the involvement of elderly individuals and those with lower levels of education. Furthermore, the nature of this study does not permit the establishment of a causal relationship between the assessed variables. The utilized sampling technique resulted in a sample that did not represent the education level of the population of Pará. However, it is important to know about access to care for COVID-19 infection in this group.

## Conclusions

Ensuring access to health services is important in a pandemic scenario. Among the sociodemographic factors that were analyzed, only sex exhibited an association with access to COVID-19 health care. The study demonstrated that people with moderate to severe COVID-19 were more likely to access health care, whereas those who used natural/alternative medicines to prevent or treat COVID-19 were less likely to access health care. Knowledge of the factors associated with access to health care for patients with pandemic diseases is crucial for organizing the health system and identifying vulnerable individuals.

## Data Availability

The datasets used during this current study are also available from the corresponding author on reasonable request.
